# Epigenetic Regulation of IL-17-Induced Chemokines in Lung Epithelial Cells

**DOI:** 10.1155/2019/9050965

**Published:** 2019-03-17

**Authors:** Jiadi Luo, Xiaojing An, Yong Yao, Carla Erb, Annabel Ferguson, Jay K. Kolls, Songqing Fan, Kong Chen

**Affiliations:** ^1^Department of Pathology, The Second Xiangya Hospital, Central South University, Changsha, Hunan, China; ^2^Division of Pulmonary Medicine, Allergy, and Critical Care Medicine, University of Pittsburgh Medical Center, Pittsburgh, PA, USA; ^3^Department of Medicine, Tulane School of Medicine, New Orleans, LA, USA

## Abstract

Epithelial cells are known to have barrier functions in multiple organs and regulate innate immune responses. Airway epithelial cells respond to IL-17 by altering their transcriptional profiles and producing antimicrobial proteins and neutrophil chemoattractants. Although IL-17 has been shown to promote inflammation through stabilizing mRNA of CXCR2 ligands, how IL-17 exerts its downstream effects on its target cells through epigenetic mechanisms is largely unknown. Using primary human bronchial epithelial cells and immortalized epithelial cell line from both human and mouse, we demonstrated that IL-17-induced CXCR2 ligand production is dependent on histone acetylation specifically through repressing HDAC5. Furthermore, the chemokine production induced by IL-17 is strictly dependent on the bromodomain and extraterminal domain (BET) family as BET inhibition abolished the IL-17A-induced proinflammatory chemokine production, indicating a pivotal role of the recognition of acetylated histones. In combination with single-cell RNA-seq analysis, we revealed that the cell lines we employed represent specific lineages and their IL-17 responses were regulated differently by the DNA methylation mechanisms. Taken together, our data strongly support that IL-17 sustains epithelial CXCR2 ligand production through epigenetic regulation and the therapeutic potential of interrupting histone modification as well as the recognition of modified histones could be evaluated in neutrophilic lung diseases.

## 1. Introduction

The IL-17 cytokine family includes 6 members, which are produced by multiple cell types [1] and signal through the IL-17 receptor family [2]. IL-17RA is shared among many IL-17 family members, while IL-17RC is the unique receptor for IL-17 and IL-17F. IL-17 and IL-17F have been demonstrated to be critical players in host defense and inflammatory diseases [3–5]. Airway epithelial cells respond to IL-17 through producing antimicrobial proteins and neutrophil chemoattractants, promoting to eradicate extracellular pathogens such as *K. pneumoniae* in the setting of host defense [6] while contributing to tissue damage and lung pathology in chronic inflammatory diseases [7].

The chemokine superfamily has expanded rapidly, since the identification of CXCL8 (IL-8) and CCL2 (MCP-1) in the late 1980s [8]. CXCR2 is mainly expressed on neutrophils and mediates neutrophil migration to sites of inflammation [9]. Several studies, including our previous work, have shown that IL-17 is a key driver for the production of these CXCR2 ligands both in vitro and in vivo [10–12]. IL-17 can promote chemokine production through mRNA stabilization and prolongation of chemokine half-life [12–15]. However, this mechanism does not explain why primary cells derived from patients with chronic inflammatory diseases spontaneously produce CXCR2 ligands without any further ex vivo stimulation [16–18]. This leads us to hypothesize that the chromatin state of these loci has been modulated to become constitutively active and this active chromatin state leads to enhanced chemokine production in these diseased settings. Indeed, such permissive chromatin structural changes in CXCR2 ligands have been observed in both skin infection [19] and lung cancer [20].

To determine if there is any epigenetic regulation in IL-17-mediated chemokine production in the lung epithelium, we took advantage of several unique inhibitors targeting various epigenetic pathways including DNA methylation and acetylated histone recognition. Our study provides novel findings on epigenetic regulation of IL-17 signaling in the lung epithelial cells and suggests an alternative epigenetic pathway to target the treatment and diagnosis of chronic inflammatory diseases.

## 2. Results

**Figure 1:**
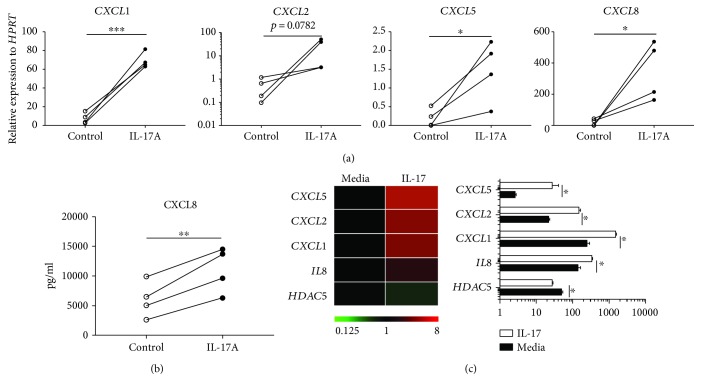
IL-17A induces proinflammatory chemokine production in primary human airway epithelial cells. (a) NHBE cells from 4 donors (derived from CORE donors) were established in the air-liquid interphase culture and treated with human recombinant IL-17A protein 100 ng/ml or control medium for 48 h. RT-PCR was performed to detect chemokine mRNA level. (b) Supernatant at 48 h was collected to measure the CXCL8 protein level by ELISA. (c) Heat map of the expression of multiple CXCR2 ligands and *HDAC5* expression in normal HBE cell cultured in air-liquid interphase in the presence or absence of 100 ng/ml IL-17 in basal media for 48 h. ^∗^ *p* < 0.05; ^∗∗^ *p* < 0.01; ^∗∗∗^ *p* < 0.001; by paired *t*-test, one-tailed.

The synergistic effects of IL-17 and TNF-*α* on the expression of IL-17-induced responses are well established [2]. To determine if IL-17 alone can induce proinflammatory chemokine production, we treated primary normal human bronchial epithelial (NHBE) cells and examined the induction of CXCR2 ligands. The data by RT-PCR and ELISA both suggested that the induction was robust and consistent among 4 different donors (Figures 1(a) and 1(b)). Since we are particularly interested in the epigenetic regulation of this induction, we mined RNA-seq data published earlier on IL-17-stimulated primary NHBE cells with the focus on these chemokines and genes that are involved in DNA methylation and histone modification. We found histone acetyltransferase (HAT) expressions were unaltered while one of the histone deacetylases, e.g., *HDAC5*, is significantly downregulated (Figure 1(c), Figure S1), suggesting that IL-17 could enhance these CXCR2 ligands through histone deacetylation. HDACs have the ability to dynamically regulate gene expression through the removal of acetyl groups from lysine residues. This process subsequently changes the chromatin accessibility and has major impacts on transcription.

**Figure 2:**
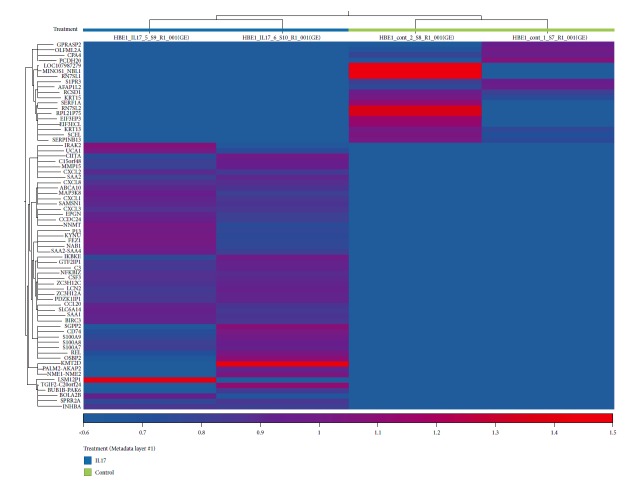
Heat map of genes regulated by IL-17A in HBE1 cells. Cells from the left two columns (under the blue bar) were treated with 100 ng/ml h-IL-17A protein for 24 h while the right two columns (under the green bar) were treated with the control medium for 24 h.

**Figure 3:**
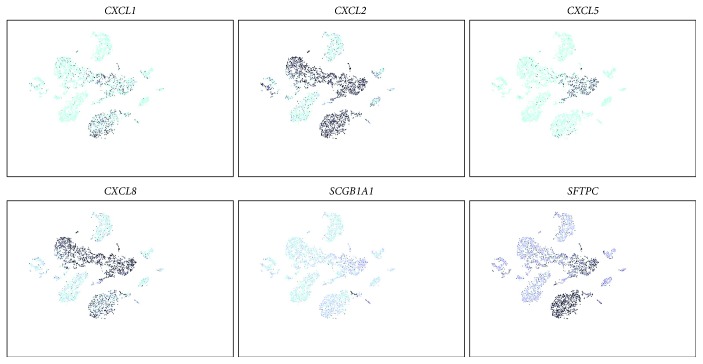
Human lung scRNA-seq. Cells were grouped into several clusters. The classification of specific cell types was inferred from the annotation of cluster-specific genes and based on expression of some well-known markers of certain cell types. *CXCL1*, *CXCL2*, *CXCL5*, *CXCL8*, *SCGB1A1*, and *SFTPC* expression cells were displayed in the cell population separately. The darker the color the cell was labelled, the higher mRNA level of target gene the cell had (the light green cells were the ones without the target gene expression).

Primary NHBE cells contain a heterogeneous population (e.g., ciliated, secretory, and basal) and could have variability in a number of responses. Therefore, to examine the underlying mechanisms by IL-17, we chose an epithelial cell line derived from a normal donor [21]. RNA-seq analysis on IL-17-treated HBE1 cells demonstrated that the gene expression profile is similar between primary NHBE cells and HBE1 cell line (Figure 2), especially in terms of the CXCR2 ligand induction including *CXCL1*, *CXCL2*, and *CXCL8*. *CXCL5* expression was extremely low in these cells, and we were unable to detect its expression by PCR (data not shown). According to our single-cell RNA-seq analysis in the normal human lung mononuclear cells, these cells had a similar gene expression profile to bronchial epithelial cells which highly express *SCGB1A1* (Figure 3). In contrast, the more type II cell-like population (marked by more *SFTPC*) seemed to be the CXCL5 producers while both populations seemed to produce other CXCR2 ligands to the same degree (Figure 3). Ingenuity Pathway Analysis (IPA) identified several top canonical pathways that were related to IL-17 signaling in a variety of cell types including the role of IL-17A in psoriasis, IL-17A signaling in fibroblasts, and differential regulation of cytokine production in intestinal epithelial cells by IL-17A and IL-17F (Figure S2A), suggesting HBE1 cells were IL-17 responsive and could be used to study signaling pathways mediated by IL-17. With the same dataset and the same upstream regulator analytic setting from the IPA software, several signaling pathways associated with innate immunity (lipopolysaccharide, TLR4) and inflammation (TNF, IL1A, and IL17C) (Figure S2B) were identified, similar to an analysis on the bronchial epithelium carried out earlier using an in vivo model [6], further proved that the HBE1 cell lines could serve as a tool for investigating the role of IL-17 in the lung epithelial cells.

**Figure 4:**
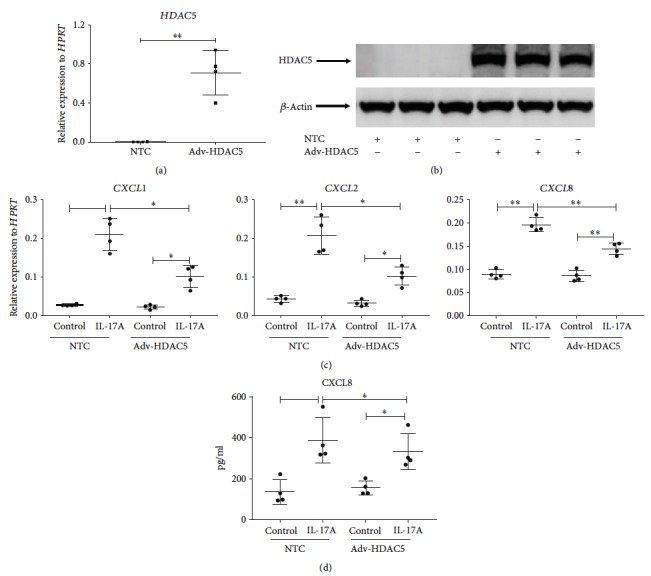
Induction of proinflammatory chemokines mediated by IL-17A can be suppressed by overexpressing HDAC5. HBE1 cells were plated 0.08-0.15 million cells per well in a 12-well plate. When 70-80% confluent, cells were transfected with 0.1 moi human HDAC5 adenovirus and nontarget adenovirus in 300 *μ*l medium, respectively, for one hour, and then we added 100 ng/ml h-IL-17A protein or 700 *μ*l control medium directly into each corresponding well in both adenovirus-infected groups. 24 h after transfection, (a) *HDAC5*, (c) *CXCL1*, *CXCL2*, and *CXCL8* mRNA levels were determined by RT-PCR. (d) 48 h supernatant after transfection was collected. CXCL8 protein level was achieved by ELISA. ^∗^ *p* < 0.05; ^∗∗^ *p* < 0.01; by paired *t*-test, two-tailed. (b) HDAC5 protein level of cells with adenovirus transfection for 24 h was detected by Western blotting (*β*-actin as reference). This experiment has been repeated.

Histone acetylation usually increases chromatin accessibility and based on the RNA-seq data from primary NHBE cells (Figure 1(c)), we decided to test if histone deacetylation would affect the IL-17 signaling by overexpressing HDAC5 using adenoviral transduction. HDAC5 overexpression was successfully achieved as assessed by PCR (Figure 4(a)) and Western blotting (Figure 4(b)). More importantly, CXCR2 ligand gene expression including *CXCL1*, *CXCL2*, and *CXCL8* was substantially repressed (Figure 4(c)), and this was also confirmed at CXCL8 protein level (Figure 4(d)).

**Figure 5:**
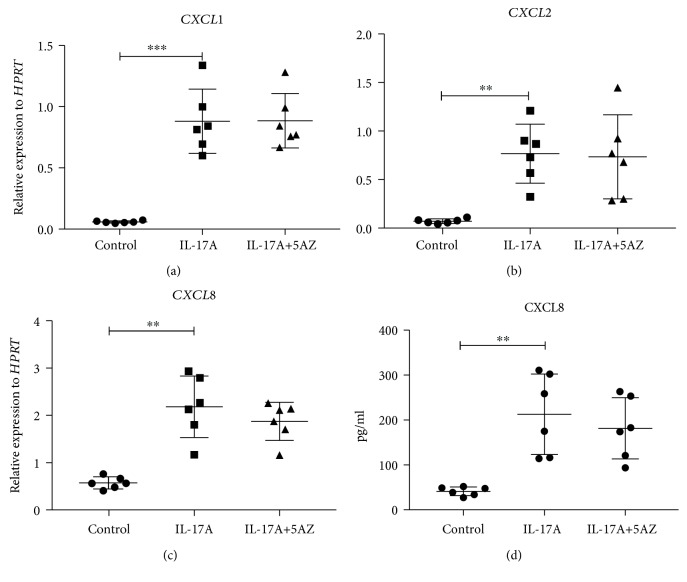
DNA methyltransferase inhibition in HBE1 cells. HBE1 cells were plated 0.08-0.15 million cells per well in a 12-well plate. After incubating with BronchiaLife™ Epithelial Airway Medium overnight, cells were treated with control medium, 100 ng/ml h-IL-17A protein, 1 *μ*M 5-azacytidine, and 100 ng/ml h-IL-17A plus 1 *μ*M 5-azacytidine, respectively. 6 h after stimulation, chemokine production (a) *CXCL1*, (b) *CXCL2*, and (c) *CXCL8* was determined by RT-PCR. (d) 24 h supernatant was collected, and CXCL8 protein level was obtained by ELISA. ^∗^ *p* < 0.05; ^∗∗^ *p* < 0.01; ^∗∗∗^ *p* < 0.001; by paired *t*-test, two-tailed. This experiment has been repeated.

**Figure 6:**
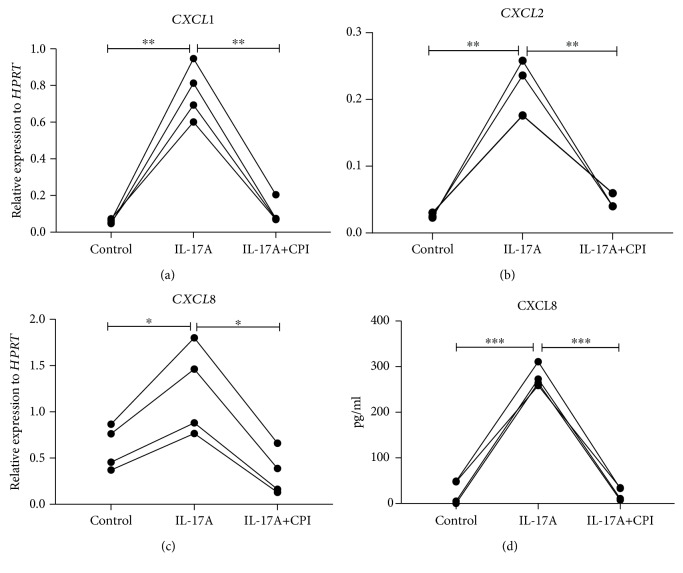
Induction of proinflammatory chemokines is dependent on BET. Human bronchial epithelial cell line HBE1 cells were plated 0.08-0.15 million cells per well in a 12-well plate. After incubating with BronchiaLife™ Epithelial Airway Medium overnight, cells were treated with control medium, 100 ng/ml h-IL-17A protein, 200 nM CPI, and 100 ng/ml h-IL-17A plus 200 nM CPI, respectively. 6 h after stimulation, (a) *CXCL1*, (b) *CXCL2*, and (c) *CXCL8* mRNA levels were determined by RT-PCR. (d) 24 h after stimulation, supernatant was collected and CXCL8 protein level was measured by ELISA. ^∗^ *p* < 0.05; ^∗∗^ *p* < 0.01; ^∗∗∗^ *p* < 0.001; by a one-way ANOVA test. It is the representative figure from 4 experiments.

**Figure 7:**
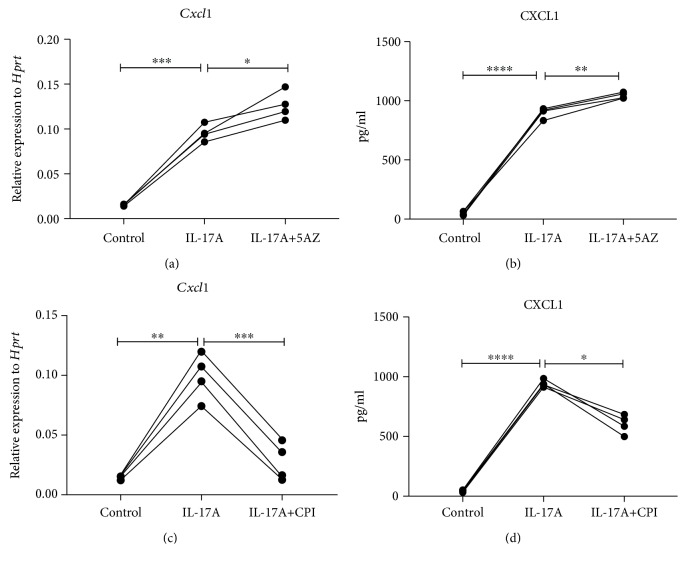
Epigenetic regulation of IL-17 pathway in mouse airway epithelial cells. Murine lung epithelial (MLE12) cells were cultured with HITES (hydrocortisone, insulin, transferrin, estradiol, and selenium) medium containing 10% fetal bovine serum (FBS) and antibiotics at 37°C in an incubator containing 5% CO_2_. Cells were treated with control medium, 50 ng/ml m-IL-17A protein, 1 *μ*M 5-azacytidine, 200 nM CPI, 50 ng/ml h-IL-17A plus 1 *μ*M 5-azacytidine and 50 ng/ml h-IL-17A plus 200 nM CPI, respectively. 6 h qPCR was performed for *Cxcl1* mRNA production with (a) IL-17A/5-azacytidine and (c) IL-17A/CPI-treated cells. 24 h supernatant was collected to reach CXCL1 protein level by ELISA for (b) IL-17A/5-azacytidine and (d) IL-17A/CPI-treated cells. ^∗^ *p* < 0.05; ^∗∗^ *p* < 0.01; ^∗∗∗^ *p* < 0.001; ^∗∗∗∗^ *p* < 0.0001; by a one-way ANOVA test. It is the representative figure from 4 experiments.

**Figure 8:**
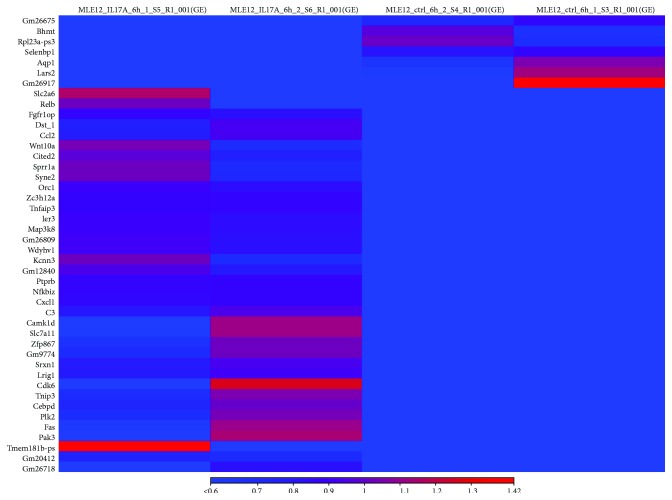
Heat map of genes regulated by IL-17A (50 ng/ml for 6 h) in MLE12 cells. The left two columns shown on the map were the two samples of m-IL-17A-treated group, and the right two columns were the control group samples.

Chromatin remodeling is well known accomplished through two main mechanisms: histone modification and DNA methylation. To explore the potential regulatory mechanism in IL-17-induced chemokine production in lung epithelial cells, we treated IL-17-stimulated HBE1 cells with 5-azacytidine (5AZ), a DNA methyltransferase inhibitor. However, inhibition DNA methyltransferase activity did not cause significant CXCR2 ligand induction or reduction in these cells, compared to single IL-17-stimulated HBE1 cells (Figure 5), suggesting that histone acetylation rather than DNA methylation is a key regulator in these cells. In contrast, when these cells were treated with a small molecule inhibitor (CPI) which blocks BET bromodomain binding, IL-17-induced CXCR2 ligand production was substantially reduced (Figure 6), suggesting an essential role of the recognition of acetylated histones in this pathway. To further investigate whether this epigenetic regulation is specific to human cells, we treated the mouse lung epithelial cell line, MLE12, with IL-17 in the presence or absence of 5AZ or CPI (Figure 7). Interestingly, CXCL1 induction was further enhanced by the inhibition of DNA methylation, suggesting that these murine cells or type II-like cells [22] can be regulated at DNA methylation level. However, BET inhibition again substantially reduced the CXCL1 expression, indicating that BET binding is essential for the induction of IL-17 downstream chemokines and this regulation is conserved in mammals. We also conducted RNA-seq analysis on IL-17-treated MLE12 cells and confirmed *CXCL1* as one of the top induced genes (Figure 8) and IPA analysis also suggested enrichment of the IL-17A and NF-*κ*B signaling pathways (Figure S3).

## 3. Discussion

IL-17 has been implicated to play essential roles in many proinflammatory lung diseases including asthma and cystic fibrosis (CF). In CF patients, chronic *Pseudomonas aeruginosa* (PA) infection leads to increased mortality by promoting irritated airway inflammation and cumulative lung damage in CF patients [23]. IL-17 levels elevated in the sputum during CF exacerbations [24], and CD4^+^ Th17 cells are identified as a critical source of IL-17 in the CF lung [25]. Indeed, PA-specific Th17 responses have been observed in the lymph nodes from patients with CF [25]. Although IL-17-mediated inflammation is essential for the clearance of extracellular pathogens such as *K. pneumoniae* and *C. albicans* [3] in several acute infection models, recent studies also suggested a possible detrimental role of the IL-17 downstream signaling in a chronic PA lung infection model through recruitment of neutrophils [26, 27]. Furthermore, HCO_3_^‑^ is indispensable for the antimicrobial function of the CF airway [28], and HCO_3_^‑^ transport can be regulated in normal human bronchial epithelial cells, however, in a cystic fibrosis transmembrane conductance regulator- (CFTR-) dependent fashion [29]. Thus, in the absence of functional CFTR, IL-17 likely contributes to pathological inflammation [3], and IL-17 itself or its downstream signaling may represent a novel target to manage the neutrophilic lung inflammation in CF. In this study, normal human and mouse cell lines were used to identify key epigenetic mechanisms of chemokine production induced by IL-17, suggesting these pathways are not unique to CF and these implications can be adapted to other lung diseases such as asthma and chronic obstructive pulmonary disease (COPD).

Epigenetic marks on histones are related to transcriptional processes. For example, trimethylated histone H3K4 is enriched at promoters [30], while monomethylated H3K4 and acetylated H3Lys27 (H3K27ac) are enriched at active enhancers [31, 32]. The bromodomain and extraterminal domain (BET) family proteins, including BRD2, BRD3, BRD4, and BRDT, contain two bromodomains, which recognize and interact with acetylated histones and other acetylated proteins with varying degrees of affinity. Small-molecule BET inhibitors mimic the acetyl moiety and insert into the bromodomain acetyl-lysine-binding pocket, which is unique to the BET family proteins. It has been shown that BRD4 plays a critical role in IL-1b-induced inflammation in human airway epithelial cells [33], and we have confirmed high levels of expression of BRD2, BRD3, and BRD4 in primary HBE cells as well as bronchial brushings obtained by clinical bronchoscopy [34], making BET inhibitors ideal candidates for blocking the constitutively active loci that have active histone marks. BET inhibition has been shown to reduce naive T cells differentiate into Th17 cells [35], consistent with the data showing that suppression of IL-17 produced by T cells isolated from CF lungs following BET inhibition. We believe that the optimal suppression of airway inflammation will be achieved by targeting both the production of IL-17 and the downstream chemokine expression. This may be critically true for chronic diseases where the genomic landscape of CXCR2 ligands is altered in the lung epithelium. Indeed, we found CXCR2 ligand production in epithelial cells can be inhibited by a BET inhibitor. However, the exact mechanism as to which histone modification yielded the inhibition needs further definition by the chromatin immunoprecipitation assay.

Primary HBE cells are heterogeneous and can be difficult to manipulate in knockdown and overexpression experiments. Thus, we used the cell lines, HBE1 and MLE12. Histone acetylation is regulated by both histone acetyltransferases (HATs) and HDAC enzymes. We did not hypothesize a regulatory mechanism by HAT as our RNA-seq data showed that HAT expression was not affected by IL-17 stimulation. However, in the experiments with HDAC overexpression, HAT can play a role to compromise the effect of altered HDAC5 expression, which may explain why we observed a modest effect using adenovirus overexpressing HDAC5 (Figure 4). Thus, the expression of HATs will be further examined by RNA-seq. HDAC5 phosphorylation and subcellular distribution have been implicated in regulating gene expression [36, 37] and could be carefully determined by Western blot in the future.

In this study, we observed major differences in DNA methylation regulation in human cells (Figure 5) vs. mouse cells (Figure 7). As to a certain gene expression, different organisms/tissues use different epigenetic machinery, so do different species. We found differential expression of Hu-antigen R (HuR), encoded by *ELAVL1*, in HBE1 and MLE12 cells (Figure S4). The ubiquitously expressed HuR protein was recently shown to regulate the expression of DNA methyltransferases posttranscriptionally [38]. Therefore, the lower expression of *ELAVL1* may explain why HBE1 cells are less sensitive to DNA methyltransferase inhibition, indicating that a tissue-specific targeting of epigenetic regulation should be considered in future drug development. The observed differences are also likely due to sequence differences in the mouse and human genome, for example, differences in CpG island distribution near the promoter regions could lead to the loci to be more resistant to DNA methylation.

Taken together, our data support IL-17 enhances chemokine production in lung epithelial cells through histone modification and recognition and the therapeutic potential of interrupting this pathway could be evaluated in IL-17-mediated diseases.

## 4. Materials and Methods

### 4.1. Primary Cell Culture and Stimulation

Human lung parenchyma tissue was processed to isolate mononuclear cells, as we previously described [34]. Cells were used for single-cell RNA sequencing (scRNA-seq) analysis.

Normal human bronchial epithelial cells (NHBE cells) obtained from the University of Pittsburgh tissue and cell core lab were prepared according to the previously described methods approved by the University of Pittsburgh IRB [39]. Cells established in the air-liquid interphase culture were exposed to 100 ng/ml human recombinant IL-17A protein (BioLegend, Cat# 570506) or control medium for 48 h. Cells were harvested in a QIAGEN RLT buffer for further gene expression detection.

### 4.2. Cell Line Culture and Stimulation

Human bronchial epithelial cell line HBE1 cells [21] were cultured in complete a bronchial epithelial airway medium (BronchiaLife™ Epithelial Airway Medium Complete Kit, Lifeline, LL-0023). Cells were plated 0.08-0.15 million cells in 1 ml medium per well in a 12-well plate. Around 80% of confluence, cells were then treated with control medium, 100 ng/ml h-IL-17A protein, 1 *μ*M 5-azacytidine (Sigma, Cat# A2385), 200 nM CPI (Cayman, Cat# 15479), 100 ng/ml h-IL-17A plus 1 *μ*M 5-azacytidine, and 100 ng/ml h-IL-17A plus 200 nM CPI, respectively, for 6 h or 24 h. At 6 h time point, cells were lysed in a RLT buffer with 2-mercaptoethanol for RNA extraction and later gene expression; at 24 h time point, supernatant was collected for ELISA. Cells stimulated with or without 100 ng/ml h-IL-17A medium for 24 h were harvested for mRNA-seq.

Murine lung epithelial cell line MLE12 cells [22] were cultured with HITES (DMEM/F12 with hydrocortisone, insulin, transferrin, estradiol, and selenium) medium containing 10% fetal bovine serum (FBS) and antibiotics at 37°C in an incubator containing 5% CO_2_. Similar to HBE1 cells, MLE12 cells were stimulated with a control medium, 50 ng/ml m-IL-17A protein (BioLegend, Cat# 576004), 1 *μ*M 5-azacytidine, 200 nM CPI, 50 ng/ml h-IL-17A plus 1 *μ*M 5-azacytidine, and 50 ng/ml h-IL-17A plus 200 nM CPI, respectively, for 6 h or 24 h. 6 h after stimulation, cells were obtained for further RNA extraction and mRNA-seq; 24 h after stimulation, supernatant was collected for protein measurement.

### 4.3. HDAC5 Overexpression

HBE1 cells were plated 0.08-0.15 million cells per well in a 12-well plate. When 70-80% confluent, cells were transfected with 0.1 moi human HDAC5 adenovirus (a.b.m., Cat# 096660A) and nontarget adenovirus (a.b.m., Cat# 000541A) in 300 *μ*l medium, respectively, for one hour, and then we added 100 ng/ml h-IL-17A protein or 700 *μ*l control medium directly into each corresponding well in both adenovirus-infected groups. 24 h after transfection, cells were harvested for protein and gene expression measurement; 48 h after transfection, supernatant was obtained for protein detection.

### 4.4. RNA Extraction and cDNA Synthesis

RNA was extracted from cell samples with RNeasy Miniprep Kit (QIAGEN, Cat# 74136; Zymo Research, Cat# R1055), according to the manufacturer's instructions. Further cDNA was constructed with qScript™ cDNA Synthesis Kits (Quantabio, Cat# 95047-100).

### 4.5. Real-Time PCR

Real-time PCR was conducted with the Bio-Rad CFX96 system employing TaqMan PCR Master Mix (Bio-Rad, Cat# 1725284) and premixed primers/probe sets (mouse: CXCL1 (Mm04207460_m1) and Hprt (Mm03024075_m1); human: CXCL1 (Hs00236937_m1), CXCL2 (Hs00601975_m1), CXCL5 (Hs01099660_g1), CXCL8 (Hs00174103_m1), HDAC5 (Hs00608351_m1), and HPRT (Hs02800695_m1)) from Thermo Fisher Scientific.

### 4.6. ELISA

ELISA kits were used for detecting human CXCL8 (BioLegend, Cat# 431505) and mouse CXCL1 (R&D Systems, Cat# DY453). The procedures were performed in strict accordance with the manufacturer's protocol.

### 4.7. Western Blotting

Equal amount of protein (30 *μ*g) was separated by Bolt™ 4-12% Bis-Tris Plus Gels (Thermo Fisher, Cat# NW04122BOX) and then electrophoretically transferred onto nitrocellulose membranes. The membranes were then blocked for 1 hour with 5% skim milk in Tris-buffered saline (TBS) Tween 20 and probed with specific primary antibodies (HDAC5: Abcam, Cat# ab55403; *β*-actin: Abcam, Cat# ab8226) at 4°C overnight. After washing the primary antibodies with TBS Tween 20, the blots were incubated with appropriate horseradish peroxidase-conjugated secondary antibodies for 2 hours at room temperature. Images were captured by ChemiDoc™ MP Imaging System (Bio-Rad, Cat# 12003154).

### 4.8. ScRNA-seq and mRNA-seq

ScRNA-seq libraries were constructed according to the “Single Cell 3' Reagent Kits v2 User Guide” (10X Genomics). Generally, single-cell population was barcoded, and barcoded cDNA was prepared inside each cell by reverse transcription. Cell lysis followed, and then cDNA library was achieved through a released barcoded cDNA amplification. Following fragmentation, end repair, and addition of a single A base, double-sided size selection was used to isolate cDNA around 200 bp. Further adaptor ligation, sample index PCR amplification, and another double-sided size selection, the final 300∼600 bp DNA sequencing library was constructed and sequenced on Illumina HiSeq by Novogene (Chula Vista, CA). The RNA-seq analysis methodology was published previously [40]. Heat maps were generated by CLC Genomics Workbench (QIAGEN Inc.).

### 4.9. Statistics

All data analyses were performed with Prism 7.0 (GraphPad). The one-way ANOVA test was used for the comparison of gene expression among the three groups. For other comparisons between the paired two groups, paired Student's *t*-test was performed.

## Data Availability

The RNA-seq data used to support the findings of this study are available from the corresponding author upon request.

## Supplementary Materials

Supplementary 1 Figure S1: heat map of the expression of multiple HDAC expression in normal HBE cell cultured in air-liquid interphase in the presence or absence of 100 ng/ml IL-17 in basal media for 48 h.

Supplementary 2 Figure S2: IPA analysis for gene expression in human bronchial epithelial cells (HBE1) cultured in BronchiaLife™ Epithelial Airway Medium, stimulated with or without 100 ng/ml IL-17A for 24 h.

Supplementary 3 Figure S3: IPA analysis for gene expression in murine lung epithelial (MLE12) cells treated with 50 ng/ml IL-17A or control medium for 6 h.

Supplementary 4 Figure S4: baseline HuR expression level in both human HBE1 and mouse airway epithelial cell line MLE12 cells. Total transcript counts of HuR from mRNA sequencing data of HBE1 and MLE12 cells without any stimulation.

Supplementary 5 Figure S5: original Western blotting membrane scan pictures. The membrane was cut into 2 parts. Two different protein markers were loaded to show the protein size (ladder labelled on the left side: ExcelBand™ 3-color Pre-Stained Protein Ladder, PM5200, SMOBIO; ladder labelled on the right side: MagicMark™ XP Western Protein Standard, LC5602, Invitrogen). Sample conditions were also listed.

## References

[1] Pappu R., Ramirez-Carrozzi V., Sambandam A. (2011). The interleukin-17 cytokine family: critical players in host defence and inflammatory diseases. *Immunology*.

[2] Gaffen S. L. (2009). Structure and signalling in the IL-17 receptor family. *Nature Reviews Immunology*.

[3] Chen K., Kolls J. K. (2013). T cell-mediated host immune defenses in the lung. *Annual Review of Immunology*.

[4] Way E. E., Chen K., Kolls J. K. (2013). Dysregulation in lung immunity - the protective and pathologic Th17 response in infection. *European Journal of Immunology*.

[5] Kumar P., Subramaniyam G. (2015). Molecular underpinnings of Th17 immune-regulation and their implications in autoimmune diabetes. *Cytokine*.

[6] Chen K., Eddens T., Trevejo-Nunez G. (2016). IL-17 receptor signaling in the lung epithelium is required for mucosal chemokine gradients and pulmonary host defense against K. pneumoniae. *Cell Host & Microbe*.

[7] Chen K., Pociask D. A., McAleer J. P. (2011). IL-17RA is required for CCL2 expression, macrophage recruitment, and emphysema in response to cigarette smoke. *PLoS One*.

[8] Zlotnik A., Yoshie O. (2012). The chemokine superfamily revisited. *Immunity*.

[9] Kobayashi Y. (2006). Neutrophil infiltration and chemokines. *Critical Reviews in Immunology*.

[10] Ye P., Rodriguez F. H., Kanaly S. (2001). Requirement of interleukin 17 receptor signaling for lung CXC chemokine and granulocyte colony-stimulating factor expression, neutrophil recruitment, and host defense. *The Journal of Experimental Medicine*.

[11] Cai S., Batra S., Lira S. A., Kolls J. K., Jeyaseelan S. (2010). CXCL1 regulates pulmonary host defense to Klebsiella infection via CXCL2, CXCL5, NF-κB, and MAPKs. *Journal of Immunology*.

[12] Herjan T., Yao P., Qian W. (2013). HuR is required for IL-17-induced Act1-mediated CXCL1 and CXCL5 mRNA stabilization. *Journal of Immunology*.

[13] Datta S., Novotny M., Pavicic P. G. (2010). IL-17 regulates CXCL1 mRNA stability via an AUUUA/tristetraprolin-independent sequence. *The Journal of Immunology*.

[14] Sun D., Novotny M., Bulek K., Liu C., Li X., Hamilton T. (2011). Treatment with IL-17 prolongs the half-life of chemokine CXCL1 mRNA via the adaptor TRAF5 and the splicing-regulatory factor SF2 (ASF). *Nature Immunology*.

[15] Hartupee J., Liu C., Novotny M., Li X., Hamilton T. (2007). IL-17 enhances chemokine gene expression through mRNA stabilization. *Journal of Immunology*.

[16] Striz I., Mio T., Adachi Y., Robbins R. A., Romberger D. J., Rennard S. I. (1999). IL-4 and IL-13 stimulate human bronchial epithelial cells to release IL-8. *Inflammation*.

[17] Gormand F., Cheria-Sammari S., Aloui R. (1995). Granulocyte-macrophage colony stimulating factors (GM-CSF) and interleukin 8 (IL-8) production by human bronchial epithelial cells (HBEC) in asthmatics and controls. Lack of in vitro effect of salbutamol compared to sodium nedocromil. *Pulmonary Pharmacology*.

[18] Takizawa H., Desaki M., Ohtoshi T. (1997). Erythromycin modulates IL-8 expression in normal and inflamed human bronchial epithelial cells. *American Journal of Respiratory and Critical Care Medicine*.

[19] Angrisano T., Pero R., Paoletti I. (2013). Epigenetic regulation of IL-8 and *β*-defensin genes in human keratinocytes in response to Malassezia furfur. *The Journal of Investigative Dermatology*.

[20] Baird A. M., Gray S. G., O'Byrne K. J. (2011). Epigenetics underpinning the regulation of the CXC (ELR+) chemokines in non-small cell lung cancer. *PLoS One*.

[21] Yankaskas J. R., Haizlip J. E., Conrad M. (1993). Papilloma virus immortalized tracheal epithelial cells retain a well-differentiated phenotype. *American Journal of Physiology-Cell Physiology*.

[22] Zhou Q., Ye X., Sun R. (2014). Differentiation of mouse induced pluripotent stem cells into alveolar epithelial cells in vitro for use in vivo. *Stem Cells Translational Medicine*.

[23] Guh A. Y., Limbago B. M., Kallen A. J. (2014). Epidemiology and prevention of carbapenem-resistant Enterobacteriaceae in the United States. *Expert Review of Anti-Infective Therapy*.

[24] McAllister F., Henry A., Kreindler J. L. (2005). Role of IL-17A, IL-17F, and the IL-17 receptor in regulating growth-related oncogene-alpha and granulocyte colony-stimulating factor in bronchial epithelium: implications for airway inflammation in cystic fibrosis. *Journal of Immunology*.

[25] Chan Y. R., Chen K., Duncan S. R. (2013). Patients with cystic fibrosis have inducible IL-17+IL-22+ memory cells in lung draining lymph nodes. *The Journal of Allergy and Clinical Immunology*.

[26] Dubin P. J., Kolls J. K. (2007). IL-23 mediates inflammatory responses to mucoid Pseudomonas aeruginosa lung infection in mice. *American Journal of Physiology-Lung Cellular and Molecular Physiology*.

[27] Dubin P. J., Martz A., Eisenstatt J. R., Fox M. D., Logar A., Kolls J. K. (2011). Interleukin-23-mediated inflammation in Pseudomonas aeruginosa pulmonary infection. *Infection and Immunity*.

[28] Pezzulo A. A., Tang X. X., Hoegger M. J. (2012). Reduced airway surface pH impairs bacterial killing in the porcine cystic fibrosis lung. *Nature*.

[29] Kreindler J. L., Bertrand C. A., Lee R. J. (2009). Interleukin-17A induces bicarbonate secretion in normal human bronchial epithelial cells. *American Journal of Physiology-Lung Cellular and Molecular Physiology*.

[30] Wang Z., Zang C., Rosenfeld J. A. (2008). Combinatorial patterns of histone acetylations and methylations in the human genome. *Nature Genetics*.

[31] Rada-Iglesias A., Bajpai R., Swigut T., Brugmann S. A., Flynn R. A., Wysocka J. (2011). A unique chromatin signature uncovers early developmental enhancers in humans. *Nature*.

[32] Shen Y., Yue F., McCleary D. F. (2012). A map of the cis-regulatory sequences in the mouse genome. *Nature*.

[33] Khan Y. M., Kirkham P., Barnes P. J., Adcock I. M. (2014). Brd4 is essential for IL-1*β*-induced inflammation in human airway epithelial cells. *PLoS One*.

[34] Chen K., Campfield B. T., Wenzel S. E. (2016). Antiinflammatory effects of bromodomain and extraterminal domain inhibition in cystic fibrosis lung inflammation. *JCI Insight*.

[35] Mele D. A., Salmeron A., Ghosh S., Huang H. R., Bryant B. M., Lora J. M. (2013). BET bromodomain inhibition suppresses TH17-mediated pathology. *The Journal of Experimental Medicine*.

[36] Wang W., Ha C. H., Jhun B. S., Wong C., Jain M. K., Jin Z. G. (2010). Fluid shear stress stimulates phosphorylation-dependent nuclear export of HDAC5 and mediates expression of KLF2 and eNOS. *Blood*.

[37] Pang J., Yan C., Natarajan K. (2008). GIT1 mediates HDAC5 activation by angiotensin II in vascular smooth muscle cells. *Arteriosclerosis, Thrombosis, and Vascular Biology*.

[38] Denis H., Ndlovu '. M. N., Fuks F. (2011). Regulation of mammalian DNA methyltransferases: a route to new mechanisms. *EMBO Reports*.

[39] Myerburg M. M., Harvey P. R., Heidrich E. M., Pilewski J. M., Butterworth M. B. (2010). Acute regulation of the epithelial sodium channel in airway epithelia by proteases and trafficking. *American Journal of Respiratory Cell and Molecular Biology*.

[40] Ray M., Horne W., McAleer J. P. (2015). RNA-seq in pulmonary medicine: how much is enough?. *American Journal of Respiratory and Critical Care Medicine*.

