# Supplementary figures and images for: Epigenetic Regulation of IL-17-Induced Chemokines in Lung Epithelial Cells
Source: Mediators Inflamm. 2019 Mar 17;2019:9050965. doi: 10.1155/2019/9050965 (PMC6441531; doi:10.1155/2019/9050965)

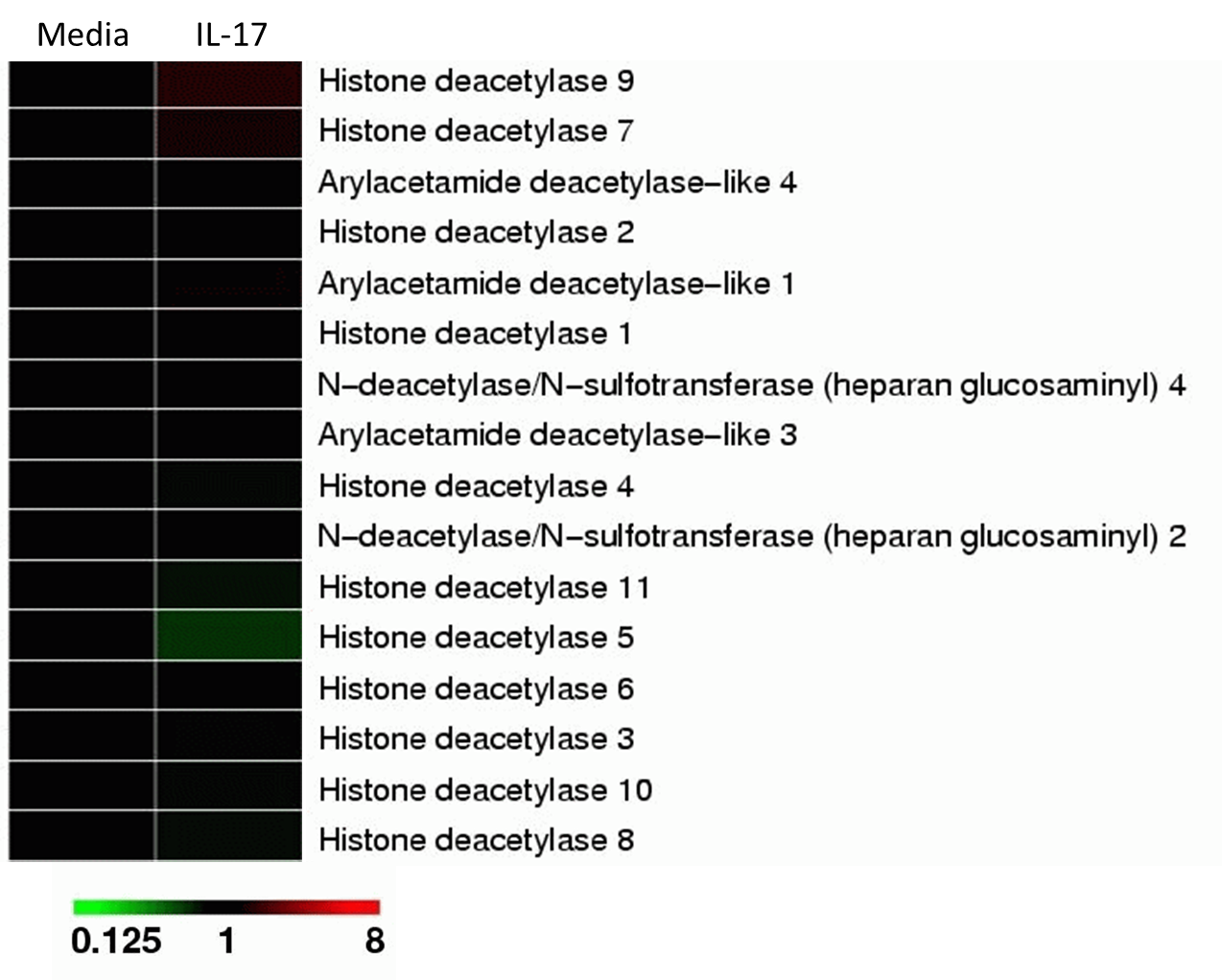

Supplement: Supplementary 1 — Figure S1: heat map of the expression of multiple HDAC expression in normal HBE cell cultured in air-liquid interphase in the presence or absence of 100 ng/ml IL-17 in basal media for 48 h. [file 9050965.f1.docx]

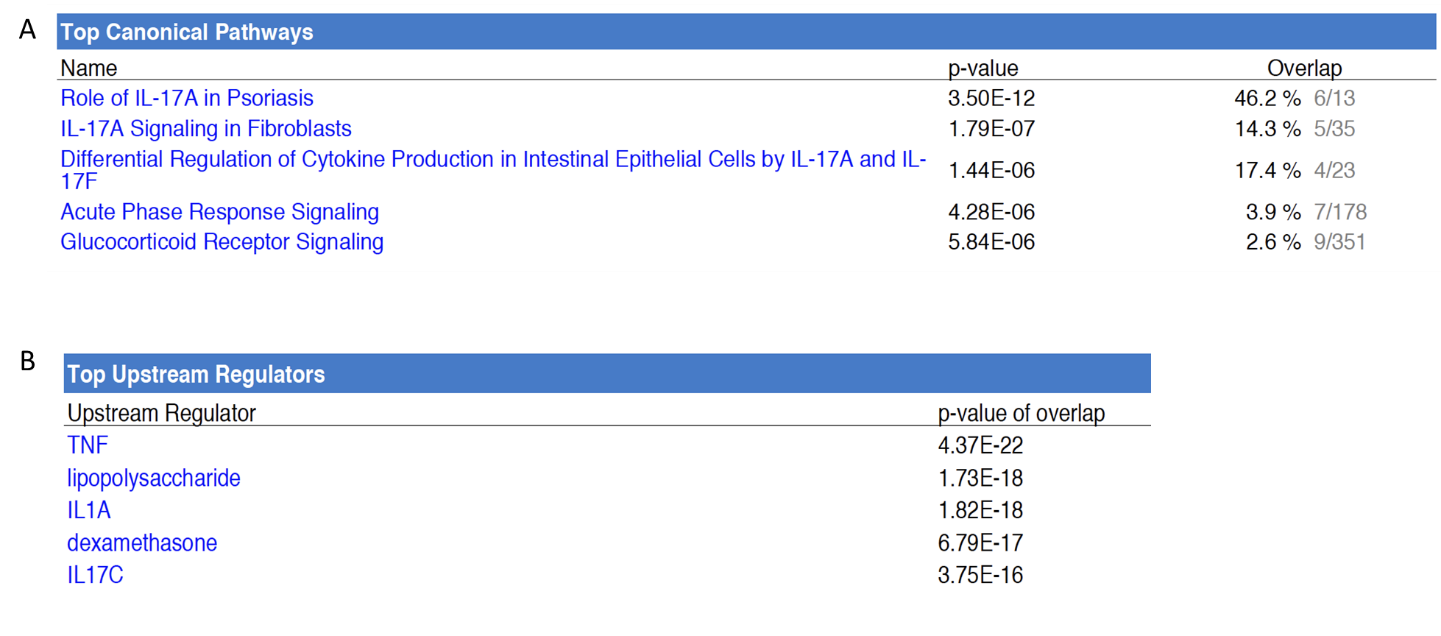

Supplement: Supplementary 2 — Figure S2: IPA analysis for gene expression in human bronchial epithelial cells (HBE1) cultured in BronchiaLife™ Epithelial Airway Medium, stimulated with or without 100 ng/ml IL-17A for 24 h. [file 9050965.f2.docx]

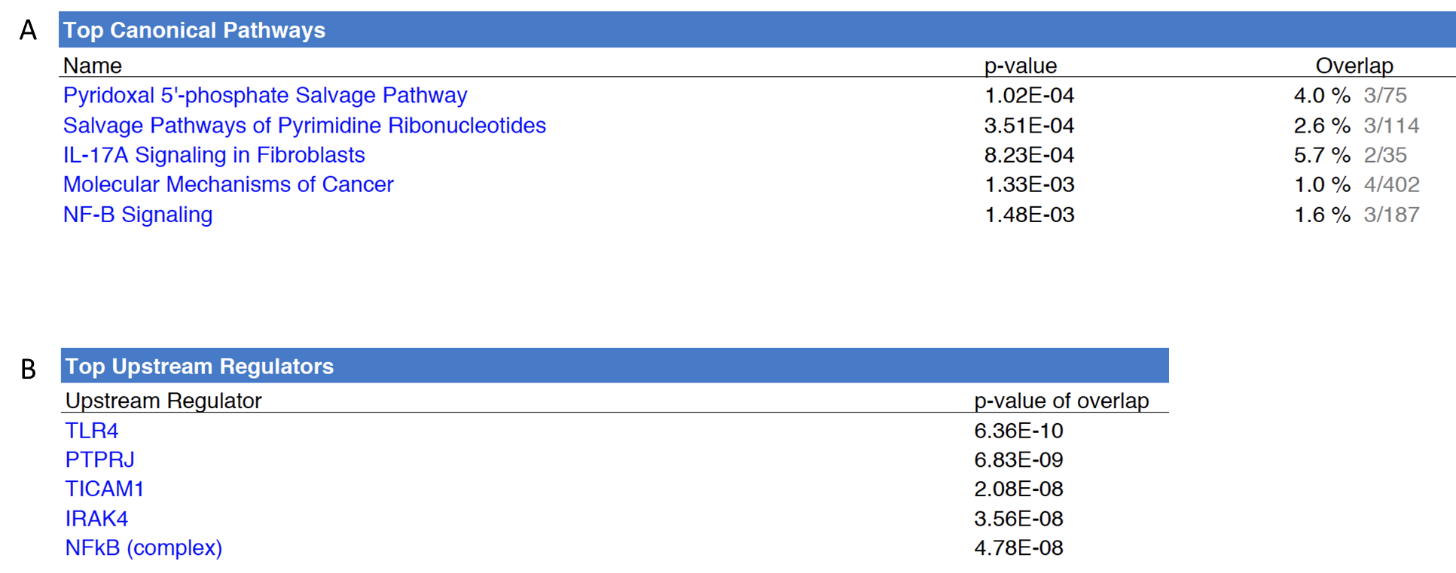

Supplement: Supplementary 3 — Figure S3: IPA analysis for gene expression in murine lung epithelial (MLE12) cells treated with 50 ng/ml IL-17A or control medium for 6 h. [file 9050965.f3.docx]

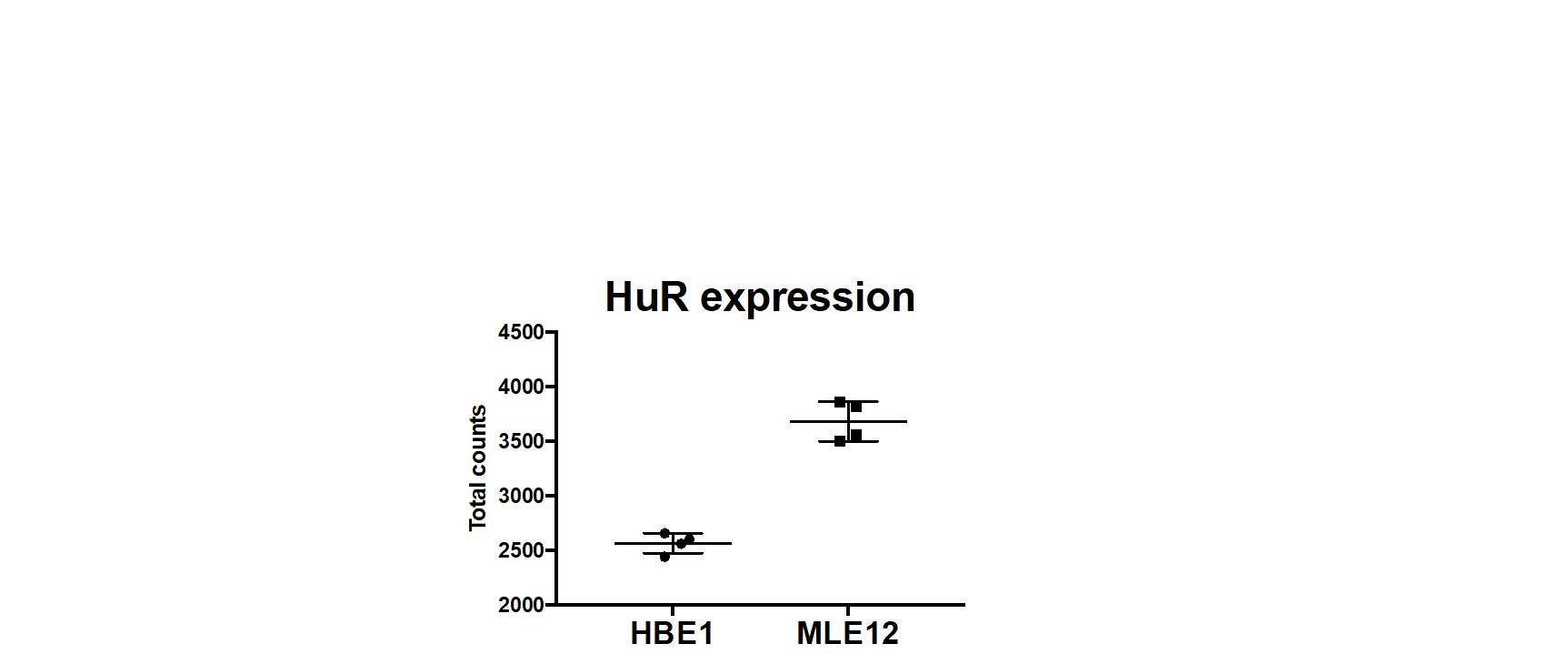

Supplement: Supplementary 4 — Figure S4: baseline HuR expression level in both human HBE1 and mouse airway epithelial cell line MLE12 cells. Total transcript counts of HuR from mRNA sequencing data of HBE1 and MLE12 cells without any stimulation. [file 9050965.f4.docx]

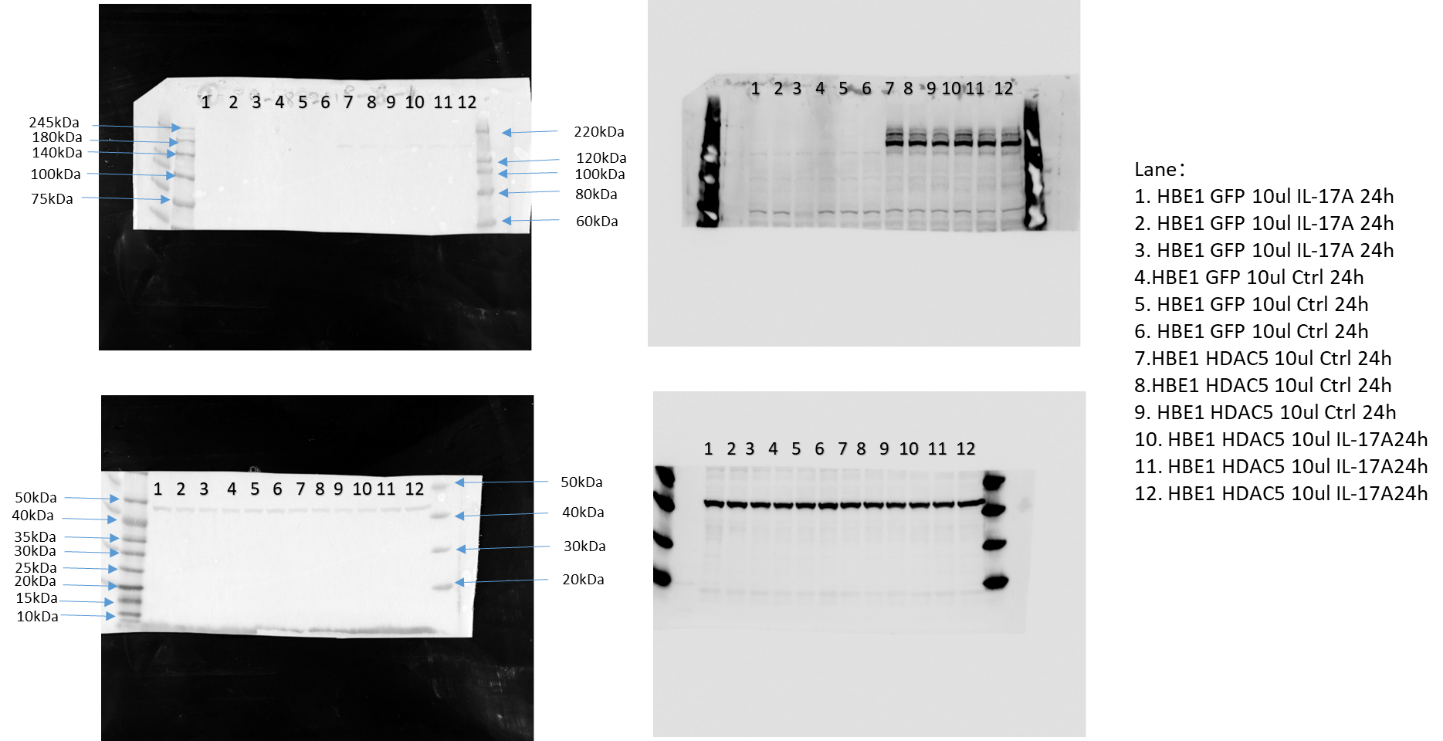

Supplement: Supplementary 5 — Figure S5: original Western blotting membrane scan pictures. The membrane was cut into 2 parts. Two different protein markers were loaded to show the protein size (ladder labelled on the left side: ExcelBand™ 3-color Pre-Stained Protein Ladder, PM5200, SMOBIO; ladder labelled on the right side: MagicMark™ XP Western Protein Standard, LC5602, Invitrogen). Sample conditions were also listed. [file 9050965.f5.docx]
